# 1,3-Bis(2-oxoprop­yl)thymine

**DOI:** 10.1107/S2414314620002576

**Published:** 2020-02-28

**Authors:** Bogdan Doboszewski, Alexander Y. Nazarenko, Fábio da Paixão Soares

**Affiliations:** a Departamento de Química, Universidade Federal Rural de Pernambuco, 52171-900 Recife, PE, Brazil; b Chemistry Department, State University of New York, College at Buffalo, 1300 Elmwood Ave, Buffalo, NY 14222-1095, USA; University of Zürich, Switzerland

**Keywords:** crystal structure, thymine, substituted nucleobase, 2-oxoprop­yl

## Abstract

In the title compound [systematic name: 5-methyl-1,3-bis­(2-oxoprop­yl)pyrimidine-2,4(1*H*,3*H*)-dione], C_11_H_14_N_2_O_4_, the two 2-oxopropyl groups are nearly perpendicular to the planar thymine unit. One methyl group of oxopropyl substituent is disordered. In the crystal, C—H⋯O inter­actions help to connect the mol­ecules into (001) layers.

## Abstract 2

In the title compound, C_11_H_14_N_2_O_4_, the two 2-oxopropyl groups are nearly perpendicular to the planar thymine unit. One methyl group of the oxopropyl substituent is disordered. In the crystal, C—H⋯O inter­actions help to connect the mol­ecules into (001) layers.

## Graphical abstract

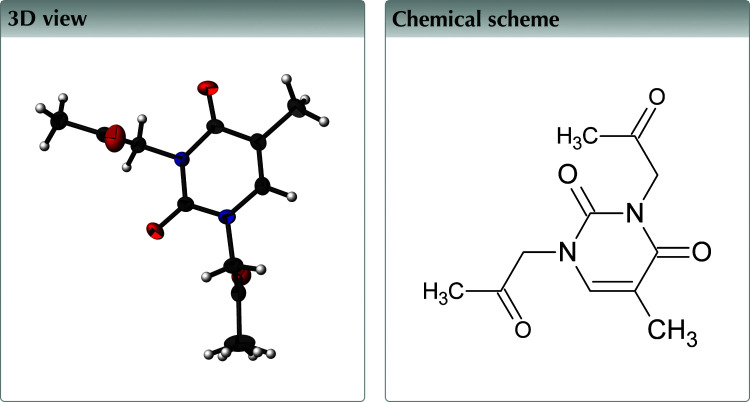

## Structure description

Nucleoside analogs play an important role in combating viral diseases and neoplasms; this is demonstrated by the pharmacological success of drugs such as Zidovudine, Stavudine, Lamivudine, and around 20 others belonging to this group of compounds (Adamska *et al.*, 2016 ▸; Krim *et al.*, 2012 ▸; Negrón-Silva *et al.*, 2013 ▸; Thakur *et al.*, 2014 ▸). We performed the transformation of *N* ^1^,*N* ^3^-bis­propargyl thymine and uracyl, which furnished *N* ^1^,*N* ^3^-bis-(2-oxopro-1-yl) derivatives; these compounds offer ample possibilities of further functionalization *via* C or O alkyl­ation of their enolates, or *via* reductive amination, among others.

**Figure 1:**
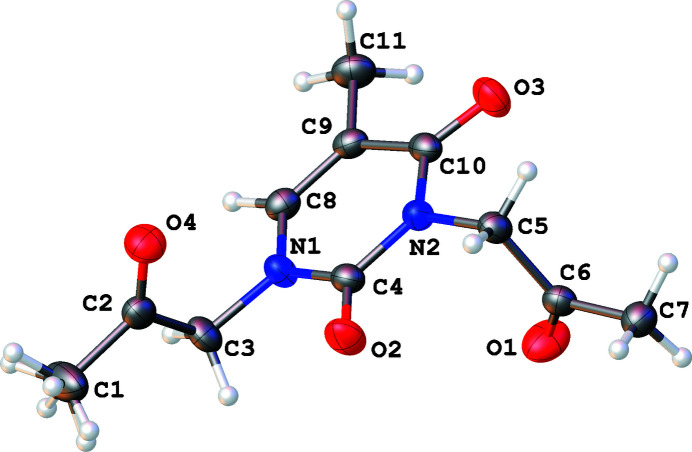
Numbering scheme for the title compound, shown with 50% probability displacement ellipsoids.

In the title compound (Fig. 1 ▸), the thymine unit is nearly planar, with the largest deviation from the mean plane being less than 0.03 Å. The two essentially planar 2-oxopropyl substituents are almost perpendicular to the thymine unit; the dihedral angles between the mean plane of the six-membered ring and those of the 2-oxopropyl fragments with atoms C1 and C7 are 77.96 (1) and 82.92 (1)°, respectively. Rotational disorder was observed for the C1 methyl group of the 2-oxopropyl substituent.

**Figure 2:**
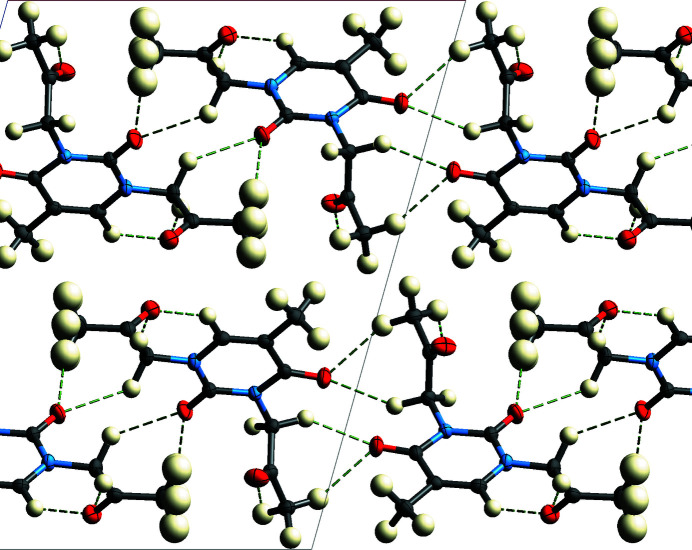
Packing of the title compound, viewed along [010].

There are no usual hydrogen bonds in this structure. Attractive C—H⋯O inter­actions (Table 1 ▸), involving all oxygen atoms of the mol­ecule, help to organize the mol­ecules in a layer parallel to the (001) plane. These layers are packed in the three-dimensional crystal by van der Waals forces, mainly between hydrogen atoms (Fig. 2 ▸).

## Synthesis and crystallization

As *N*1,*N*3-bis­propargyl thymine can be prepared in yields exceeding 80%, we used it as a starting material to obtain the title compound (**1**) *via* addition of water catalyzed by silica-supported HgSO_4_/H_2_SO_4_ (Mello *et al.*, 2010 ▸). This furnished the necessary **1** (*R*_f_ 0.30, EtOAc neat, more polar than *N*1*,N*3-bis-propargylthymine, *R*_f_ 0.70, hexane-EtOAc) by simple filtration of the solids and crystallization from ethyl acetate. The product formed well-resolved crystals suitable for X-ray analysis. Compound **1**: m.p. 408–412 K (EtOAc). ^1^H NMR (300 MHz, DMSO-*d*_6_): 7.47 (broadened *s*, 1H, H6), 4.66 and 4.64 (two *s*, total of 4H, NCH_2_), 2.16 and 2.14 (two *s*, total of 6H, COCH_3_), 1.80 (broadened *s*, 3H, C5CH_3_). ^13^C NMR (75 MHz, DMSO-*d*_6_): 201.8 and 201.3 (two **C**OCH_3_), 162.0 and 150.6 (C2, 4), 140.8 (C6), 107.3 (C5), 56.9 and 49.7 (two CH_2_), 27.1 and 26.8 (two CO**C**H_3_), 12.4 (C5**C**H_3_).

## Refinement

Crystal data, data collection and structure refinement details are summarized in Table 2 ▸. Methyl group C1 is disordered.

## Supplementary Material

Crystal structure: contains datablock(s) I. DOI: 10.1107/S2414314620002576/zq4039sup1.cif

Structure factors: contains datablock(s) I. DOI: 10.1107/S2414314620002576/zq4039Isup2.hkl

Supporting information file. DOI: 10.1107/S2414314620002576/zq4039Isup3.cdx

Supporting information file. DOI: 10.1107/S2414314620002576/zq4039Isup4.cml

CCDC reference: 1986083

Additional supporting information:  crystallographic information; 3D view; checkCIF report

## Figures and Tables

**Table 1:** Hydrogen-bond geometry (Å, °)

*D*—H⋯*A*	*D*—H	H⋯*A*	*D*⋯*A*	*D*—H⋯*A*
C3—H3*A*⋯O2^i^	0.97 (1)	2.56 (1)	3.304 (2)	134 (1)
C3—H3*B*⋯O4^ii^	0.97 (1)	2.45 (1)	3.3820 (19)	160 (1)
C5—H5*B*⋯O3^iii^	0.96 (1)	2.50 (1)	3.353 (2)	148 (1)
C7—H7*A*⋯O1^iv^	0.96 (1)	2.54 (1)	3.2576 (19)	131 (1)
C7—H7*B*⋯O3^iii^	0.96 (1)	2.50 (1)	3.370 (2)	150 (1)
C8—H8⋯O4^ii^	0.96 (1)	2.50 (1)	3.3213 (19)	144 (1)

Symmetry codes: (i) 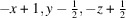 ; (ii) 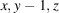 ; (iii) 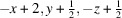 ; (iv) 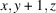 .

**Table 2:** Experimental details

Crystal data	
Chemical formula	C_11_H_14_N_2_O_4_
*M*_r_	238.24
Crystal system, space group	Monoclinic, *P*2_1_/*c*
Temperature (K)	173
*a*, *b*, *c* (Å)	13.9490 (9), 4.9891 (3), 17.3647 (11)
β (°)	105.693 (4)
*V* (Å^3^)	1163.41 (13)
*Z*	4
Radiation type	Mo *K*α
μ (mm^−1^)	0.11
Crystal size (mm)	0.2 × 0.15 × 0.08

Data collection	
Diffractometer	Bruker PHOTON-100 CMOS
Absorption correction	Multi-scan (*SADABS*; Krause *et al.*, 2015 ▸)
*T*_min_, *T*_max_	0.769, 0.862
No. of measured, independent and observed [*I* > 2σ(*I*)] reflections	18736, 2458, 1889
*R*_int_	0.051
(sin θ/λ)_max_ (Å^−1^)	0.632

Refinement	
*R*[*F* ^2^ > 2σ(*F* ^2^)], *wR*(*F* ^2^), *S*	0.042, 0.103, 1.03
No. of reflections	2458
No. of parameters	163
H-atom treatment	H atoms treated by a mixture of independent and constrained refinement
Δρ_max_, Δρ_min_ (e Å^−3^)	0.21, −0.19

Computer programs: *APEX2* (Bruker, 2013 ▸) *SAINT* (Bruker, 2016 ▸), *SHELXT* (Sheldrick, 2015*a* ▸), *SHELXL* (Sheldrick, 2015*b* ▸) and *OLEX2* (Dolomanov *et al.*, 2009 ▸).

## full crystallographic data

### Crystal data

**Table:**

C_11_H_14_N_2_O_4_	*D*_x_ = 1.360 Mg m^−^^3^
*M**_r_* = 238.24	Melting point: 412 K
Monoclinic, *P*2_1_/*c*	Mo *K*α radiation, λ = 0.71073 Å
*a* = 13.9490 (9) Å	Cell parameters from 2683 reflections
*b* = 4.9891 (3) Å	θ = 3.0–25.8°
*c* = 17.3647 (11) Å	µ = 0.11 mm^−^^1^
β = 105.693 (4)°	*T* = 173 K
*V* = 1163.41 (13) Å^3^	Plate, colourless
*Z* = 4	0.2 × 0.15 × 0.08 mm
*F*(000) = 504	

### Data collection

**Table:**

Bruker PHOTON-100 CMOS diffractometer	2458 independent reflections
Radiation source: sealedtube	1889 reflections with *I* > 2σ(*I*)
Detector resolution: 10.4 pixels mm^-1^	*R*_int_ = 0.051
ω scans	θ_max_ = 26.7°, θ_min_ = 3.0°
Absorption correction: multi-scan (SADABS; Krause *et al.*, 2015)	*h* = −17→17
*T*_min_ = 0.769, *T*_max_ = 0.862	*k* = −6→6
18736 measured reflections	*l* = −21→21

### Refinement

**Table:**

Refinement on *F*^2^	Primary atom site location: dual
Least-squares matrix: full	Secondary atom site location: difference Fourier map
*R*[*F*^2^ > 2σ(*F*^2^)] = 0.042	Hydrogen site location: inferred from neighbouring sites
*wR*(*F*^2^) = 0.103	H atoms treated by a mixture of independent and constrained refinement
*S* = 1.03	*w* = 1/[σ^2^(*F*_o_^2^) + (0.0421*P*)^2^ + 0.5355*P*] where *P* = (*F*_o_^2^ + 2*F*_c_^2^)/3
2458 reflections	(Δ/σ)_max_ < 0.001
163 parameters	Δρ_max_ = 0.21 e Å^−^^3^
0 restraints	Δρ_min_ = −0.18 e Å^−^^3^

### Special details

**Table:**

Geometry. All esds (except the esd in the dihedral angle between two l.s. planes) are estimated using the full covariance matrix. The cell esds are taken into account individually in the estimation of esds in distances, angles and torsion angles; correlations between esds in cell parameters are only used when they are defined by crystal symmetry. An approximate (isotropic) treatment of cell esds is used for estimating esds involving l.s. planes.
Refinement. Methylene hydrogen atoms are refined with riding coordinates and with *U*_iso_(H) = 1.2 *U*_iso_(C); methyl hydrogen atoms are refined as rotating idealized methyl groups and with *U*_iso_(H) = 1.5 *U*_iso_(C).

### Fractional atomic coordinates and isotropic or equivalent isotropic displacement parameters (Å^2^)

**Table:**

	*x*	*y*	*z*	*U*_iso_*/*U*_eq_	Occ. (<1)
O1	0.83624 (10)	0.9143 (2)	0.36977 (7)	0.0433 (3)	
O2	0.63791 (8)	1.2059 (2)	0.24695 (7)	0.0361 (3)	
O3	0.92187 (8)	0.9349 (2)	0.18756 (7)	0.0378 (3)	
O4	0.50121 (8)	1.2301 (2)	0.06408 (7)	0.0364 (3)	
N1	0.62809 (9)	0.8661 (3)	0.15811 (7)	0.0258 (3)	
N2	0.78007 (9)	1.0765 (3)	0.21574 (7)	0.0244 (3)	
C1	0.35466 (13)	1.0758 (5)	0.09457 (13)	0.0579 (6)	
H1A	0.333498	1.262010	0.097365	0.087*	0.43 (3)
H1B	0.343268	0.974481	0.139636	0.087*	0.43 (3)
H1C	0.316192	0.995262	0.044202	0.087*	0.43 (3)
H1D	0.328474	0.892492	0.090103	0.087*	0.57 (3)
H1E	0.318704	1.180021	0.047833	0.087*	0.57 (3)
H1F	0.345780	1.159240	0.143267	0.087*	0.57 (3)
C2	0.46300 (11)	1.0691 (3)	0.09839 (9)	0.0285 (4)	
C3	0.52141 (11)	0.8459 (3)	0.14837 (10)	0.0291 (4)	
H3A	0.50906 (19)	0.8483 (3)	0.2009 (7)	0.035*	
H3B	0.4978 (3)	0.675 (2)	0.1234 (3)	0.035*	
C4	0.67906 (11)	1.0595 (3)	0.20955 (9)	0.0252 (3)	
C5	0.83796 (11)	1.2601 (3)	0.27622 (8)	0.0261 (3)	
H5A	0.8008 (5)	1.422 (2)	0.27574 (8)	0.031*	
H5B	0.8985 (8)	1.3054 (7)	0.26324 (18)	0.031*	
C6	0.86268 (11)	1.1388 (3)	0.35899 (9)	0.0266 (3)	
C7	0.91998 (12)	1.3150 (3)	0.42473 (9)	0.0316 (4)	
H7A	0.8774 (5)	1.456 (2)	0.4341 (5)	0.047*	
H7B	0.9756 (8)	1.392 (2)	0.4098 (3)	0.047*	
H7C	0.9440 (8)	1.2108 (12)	0.4727 (6)	0.047*	
C8	0.67438 (11)	0.7030 (3)	0.11540 (9)	0.0259 (3)	
H8	0.6350 (7)	0.574 (2)	0.0796 (7)	0.031*	
C9	0.77188 (11)	0.7166 (3)	0.12144 (8)	0.0250 (3)	
C10	0.83202 (11)	0.9100 (3)	0.17581 (9)	0.0254 (3)	
C11	0.82282 (13)	0.5375 (4)	0.07549 (10)	0.0347 (4)	
H11A	0.7752 (6)	0.398 (2)	0.0469 (7)	0.052*	
H11B	0.8811 (8)	0.449 (2)	0.1132 (4)	0.052*	
H11C	0.8461 (8)	0.6461 (12)	0.0358 (6)	0.052*	

### Atomic displacement parameters (Å^2^)

**Table:**

	*U*^11^	*U*^22^	*U*^33^	*U*^12^	*U*^13^	*U*^23^
O1	0.0613 (9)	0.0324 (7)	0.0357 (7)	−0.0108 (6)	0.0122 (6)	0.0031 (5)
O2	0.0298 (6)	0.0419 (7)	0.0409 (7)	−0.0006 (5)	0.0169 (5)	−0.0137 (6)
O3	0.0215 (6)	0.0471 (8)	0.0452 (7)	0.0015 (5)	0.0095 (5)	−0.0046 (6)
O4	0.0377 (7)	0.0335 (7)	0.0383 (7)	−0.0051 (5)	0.0108 (5)	0.0024 (5)
N1	0.0200 (6)	0.0270 (7)	0.0309 (7)	−0.0016 (5)	0.0079 (5)	−0.0025 (6)
N2	0.0212 (6)	0.0294 (7)	0.0234 (6)	−0.0016 (5)	0.0073 (5)	−0.0028 (5)
C1	0.0299 (10)	0.0888 (17)	0.0564 (12)	0.0140 (10)	0.0142 (9)	0.0332 (12)
C2	0.0279 (8)	0.0338 (9)	0.0241 (7)	−0.0036 (7)	0.0075 (6)	−0.0053 (7)
C3	0.0213 (8)	0.0303 (9)	0.0366 (9)	−0.0047 (6)	0.0093 (6)	−0.0001 (7)
C4	0.0221 (7)	0.0288 (8)	0.0260 (7)	−0.0009 (6)	0.0086 (6)	0.0001 (6)
C5	0.0242 (8)	0.0285 (8)	0.0255 (8)	−0.0042 (6)	0.0064 (6)	−0.0023 (6)
C6	0.0237 (8)	0.0296 (8)	0.0278 (8)	0.0008 (6)	0.0091 (6)	0.0004 (7)
C7	0.0313 (9)	0.0361 (9)	0.0262 (8)	−0.0022 (7)	0.0058 (6)	0.0011 (7)
C8	0.0302 (8)	0.0248 (8)	0.0227 (7)	−0.0013 (6)	0.0074 (6)	−0.0010 (6)
C9	0.0295 (8)	0.0249 (8)	0.0217 (7)	0.0038 (6)	0.0087 (6)	0.0028 (6)
C10	0.0222 (8)	0.0304 (8)	0.0246 (7)	0.0038 (6)	0.0081 (6)	0.0050 (6)
C11	0.0383 (9)	0.0361 (10)	0.0340 (9)	0.0054 (8)	0.0168 (7)	−0.0022 (8)

### Geometric parameters (Å, º)

**Table:**

O1—C6	1.2093 (19)	C2—C3	1.508 (2)
O2—C4	1.2191 (18)	C3—H3A	0.972 (12)
O3—C10	1.2205 (18)	C3—H3B	0.972 (12)
O4—C2	1.2066 (19)	C5—H5A	0.958 (12)
N1—C3	1.4551 (19)	C5—H5B	0.958 (12)
N1—C4	1.3749 (19)	C5—C6	1.511 (2)
N1—C8	1.3742 (19)	C6—C7	1.491 (2)
N2—C4	1.3864 (18)	C7—H7A	0.962 (10)
N2—C5	1.4612 (19)	C7—H7B	0.962 (10)
N2—C10	1.4027 (19)	C7—H7C	0.962 (10)
C1—H1A	0.9800	C8—H8	0.959 (17)
C1—H1B	0.9800	C8—C9	1.337 (2)
C1—H1C	0.9800	C9—C10	1.449 (2)
C1—H1D	0.9800	C9—C11	1.499 (2)
C1—H1E	0.9800	C11—H11A	0.997 (11)
C1—H1F	0.9800	C11—H11B	0.997 (11)
C1—C2	1.495 (2)	C11—H11C	0.997 (11)
			
C4—N1—C3	117.21 (12)	H3A—C3—H3B	107.8
C8—N1—C3	120.74 (13)	O2—C4—N1	122.08 (13)
C8—N1—C4	122.02 (12)	O2—C4—N2	122.48 (14)
C4—N2—C5	116.53 (12)	N1—C4—N2	115.44 (13)
C4—N2—C10	125.13 (13)	N2—C5—H5A	109.2
C10—N2—C5	117.87 (12)	N2—C5—H5B	109.2
H1A—C1—H1B	109.5	N2—C5—C6	111.84 (13)
H1A—C1—H1C	109.5	H5A—C5—H5B	107.9
H1A—C1—H1D	141.1	C6—C5—H5A	109.2
H1A—C1—H1E	56.3	C6—C5—H5B	109.2
H1A—C1—H1F	56.3	O1—C6—C5	121.18 (14)
H1B—C1—H1C	109.5	O1—C6—C7	123.40 (14)
H1B—C1—H1D	56.3	C7—C6—C5	115.42 (13)
H1B—C1—H1E	141.1	C6—C7—H7A	109.5
H1B—C1—H1F	56.3	C6—C7—H7B	109.5
H1C—C1—H1D	56.3	C6—C7—H7C	109.5
H1C—C1—H1E	56.3	H7A—C7—H7B	109.5
H1C—C1—H1F	141.1	H7A—C7—H7C	109.5
H1D—C1—H1E	109.5	H7B—C7—H7C	109.5
H1D—C1—H1F	109.5	N1—C8—H8	118.5
H1E—C1—H1F	109.5	C9—C8—N1	122.96 (14)
C2—C1—H1A	109.5	C9—C8—H8	118.5
C2—C1—H1B	109.5	C8—C9—C10	118.83 (14)
C2—C1—H1C	109.5	C8—C9—C11	123.13 (14)
C2—C1—H1D	109.5	C10—C9—C11	118.03 (13)
C2—C1—H1E	109.5	O3—C10—N2	120.12 (14)
C2—C1—H1F	109.5	O3—C10—C9	124.33 (14)
O4—C2—C1	122.64 (16)	N2—C10—C9	115.55 (12)
O4—C2—C3	122.26 (14)	C9—C11—H11A	109.5
C1—C2—C3	115.10 (14)	C9—C11—H11B	109.5
N1—C3—C2	113.07 (13)	C9—C11—H11C	109.5
N1—C3—H3A	109.0	H11A—C11—H11B	109.5
N1—C3—H3B	109.0	H11A—C11—H11C	109.5
C2—C3—H3A	109.0	H11B—C11—H11C	109.5
C2—C3—H3B	109.0		
			
O4—C2—C3—N1	6.9 (2)	C5—N2—C4—O2	6.5 (2)
N1—C8—C9—C10	0.5 (2)	C5—N2—C4—N1	−173.64 (13)
N1—C8—C9—C11	179.45 (14)	C5—N2—C10—O3	−5.4 (2)
N2—C5—C6—O1	0.6 (2)	C5—N2—C10—C9	175.01 (12)
N2—C5—C6—C7	−178.80 (12)	C8—N1—C3—C2	−103.59 (16)
C1—C2—C3—N1	−173.11 (16)	C8—N1—C4—O2	179.34 (15)
C3—N1—C4—O2	1.3 (2)	C8—N1—C4—N2	−0.5 (2)
C3—N1—C4—N2	−178.54 (13)	C8—C9—C10—O3	178.02 (15)
C3—N1—C8—C9	179.04 (14)	C8—C9—C10—N2	−2.5 (2)
C4—N1—C3—C2	74.43 (17)	C10—N2—C4—O2	178.41 (14)
C4—N1—C8—C9	1.1 (2)	C10—N2—C4—N1	−1.7 (2)
C4—N2—C5—C6	79.50 (16)	C10—N2—C5—C6	−93.04 (15)
C4—N2—C10—O3	−177.28 (14)	C11—C9—C10—O3	−1.0 (2)
C4—N2—C10—C9	3.2 (2)	C11—C9—C10—N2	178.53 (13)

### Hydrogen-bond geometry (Å, º)

**Table:**

*D*—H···*A*	*D*—H	H···*A*	*D*···*A*	*D*—H···*A*
C3—H3*A*···O2^i^	0.97 (1)	2.56 (1)	3.304 (2)	134 (1)
C3—H3*B*···O4^ii^	0.97 (1)	2.45 (1)	3.3820 (19)	160 (1)
C5—H5*B*···O3^iii^	0.96 (1)	2.50 (1)	3.353 (2)	148 (1)
C7—H7*A*···O1^iv^	0.96 (1)	2.54 (1)	3.2576 (19)	131 (1)
C7—H7*B*···O3^iii^	0.96 (1)	2.50 (1)	3.370 (2)	150 (1)
C8—H8···O4^ii^	0.96 (1)	2.50 (1)	3.3213 (19)	144 (1)

Symmetry codes: (i) −*x*+1, *y*−1/2, −*z*+1/2; (ii) *x*, *y*−1, *z*; (iii) −*x*+2, *y*+1/2, −*z*+1/2; (iv) *x*, *y*+1, *z*.
